# The value of testing for *ATXN2* intermediate repeat expansions in routine clinical practice for amyotrophic lateral sclerosis

**DOI:** 10.1038/s41431-022-01146-2

**Published:** 2022-07-21

**Authors:** Kristiana Salmon, Jay P. Ross, Vanessa Bertone, Maria Gobbo, Nancy Anoja, Jason Karamchandani, Patrick A. Dion, Guy A. Rouleau, Angela Genge

**Affiliations:** 1 Department of Neurology and Neurosurgery, McGill University, Montreal, QC Canada; 2 Montreal Neurological Institute and Hospital, McGill University, Montreal, QC Canada; 3 Department of Human Genetics, McGill University, Montreal, QC Canada; 4 Department of Medical Genetics, McGill University Health Centre, Montreal, QC Canada; 5 Department of Pathology, McGill University, Montreal, QC Canada

**Keywords:** Genetic testing, Genetic testing

## Introduction

Amyotrophic lateral sclerosis (ALS) is genetically diverse, with numerous variants associated with either causing or increasing risk of developing the condition [1]. The increase in genetically-targeted therapies in development, combined with a greater appreciation for the role genetics plays in ALS, has led to a push for more widespread genetic testing as part of routine clinical care [2].

Ataxin-2, a polyglutamine (polyQ) protein, is a modifier of TDP-43 toxicity in ALS models, and intermediate polyQ repeats in the *ATXN2* gene have been associated with increased susceptibility for ALS [3, 4]. An antisense oligonucleotide targeting *ATXN2* is currently in a Phase 1 clinical trial for people living with ALS, both with and without polyQ expansions (NCT04494256). To date, studies report varying incidence of *ATXN2* intermediate repeats in ALS patients [5]. With *ATXN2* repeat expansion testing now available on several commercial ALS testing panels, implementation of *ATXN2* genetic testing provides an opportunity to identify suitable participants for clinical trials.

Here, we outline our clinic’s early experience with implementing *ATXN2* genetic testing as part of routine clinical practice.

## Implementing *ATXN2* genetic testing in a clinical setting

The ALS clinic at the Montreal Neurological Institute-Hospital (The Neuro) has been offering genetic testing to all newly diagnosed ALS patients since 2015. A tiered testing approach is used—for patients with a family history of ALS, FTD, related disorders, or an atypical presentation (such as a young age of onset), a comprehensive panel of ALS genes is ordered; and, for isolated ALS, only those genes for which there are targeted, ongoing clinical trials are ordered. With this testing algorithm, both *SOD1* and *C9orf72* testing have been consistently offered to all patients for several years. Initiation of a clinical trial targeting *ATXN2* intermediate repeat expansions prompted an evolution of the clinic’s genetic testing practices to include offering *ATXN2* testing to all patients.

Clinical genetic testing at The Neuro’s ALS clinic is performed by PreventionGenetics, who added *ATXN2* testing to their offering in October 2020. Subsequently, all patients newly diagnosed with ALS would be offered *ATXN2* repeat expansion testing as part of the clinic’s standard practice. To date, 61 newly diagnosed ALS patients have had *ATXN2* genetic testing.

## Screening for *ATXN2* intermediate repeat expansions

For previously diagnosed patients, for whom genetic testing was completed prior to October 2020, repeat testing would consume considerable clinical and financial resources. However, patients at The Neuro’s ALS clinic are given the opportunity to consent to the Clinical Biospecimen Imaging and Genetic (C-BIG) Repository, where their DNA is banked for future analysis and scientific research. A request was made to C-BIG to access samples from ALS patients actively being followed in the clinic (*n* = 116). This request was approved by their Tissue and Data Committee under the ethical framework approved by the McGill University Health Centre’s Research Ethics Board—Neuroscience and Psychiatry Panel (MUHC-15-944 (CRU)/2017-330, 15-944-MUHC).

DNA was extracted from blood samples using standard procedures. The *ATXN2* CAG repeat region was amplified following published methodology [6], with minor changes (M13 universal sequence not added to primers). For 11 samples, the repeat was estimated from whole genome sequencing data using ExpansionHunter v3.2.0 [7]. Repeat alleles were considered “intermediate” length with at least 27 repeats.

## Characteristics of patients with intermediate *ATXN2* repeats

Since adding *ATXN2* intermediate repeat expansion testing to the clinic’s standard genetic testing practices, five patients have been identified (Table 1): four were identified through the retrospective screening of banked DNA samples, and one newly diagnosed patient was identified through prospective clinical testing. Consistent with previous studies [5], none of the patients had a family history of ALS, or related disorders, further emphasising the need to offer genetic testing to all people living with ALS.

**Table 1:** Clinical features of ALS patients with intermediate repeats of *ATXN2*.

	Gender	Age at symptom onset	Site of onset	Disease duration (months)	Family history of ALS, FTD, or related disorder?	Repeat length (A1/A2)
1^a^	M	44	Limb Lower extremity	41	None	22/31
2	F	38	Limb Lower extremity	48	None	22/31
3^a^	F	72	Limb Lower extremity	13	None	22/27
4	F	70	Limb Upper extremity	33	None	22/29
5	M	65	Limb Lower extremity	20	None	22/32

Five patients with intermediate repeats of the *ATXN2* gene were identified from a total sample of 177 patients, for a yield of 2.8% (retrospective screen *n* = 116; prospective clinical genetic testing *n* = 61).

*A1/A2* allele 1/allele 2.

^a^Indicates patient is deceased at time of data collection.

## Genetic counselling implications for *ATXN2*

Intermediate repeats in *ATXN2* are associated with increased risk of developing ALS, as opposed to a monogenetic, inherited cause. Should a test result indicate that intermediate repeats are present, this introduces challenges in conveying the risk to family members in post-test counselling, as the risk and implications are unclear. Genetic counselling, provided by an appropriate specialist, should always accompany the offer of genetic testing for people living with ALS.

## Value of *ATXN2* testing in clinical practice

Genetics has long contributed to our understanding of ALS, and is translating into the development of targeted therapies at an increasing rate. As such, genetic testing practices in ALS need to evolve with emerging clinical trials. Implementation of routine *ATNX2* testing in our clinical setting identified people living with ALS who were potentially eligible for a targeted genetic therapy trial. Use of banked DNA samples to conduct a retrospective screen of those patients who had previously had genetic testing reduced the burden on both financial and human resources, in our public healthcare setting. This model could be applied in other clinical settings, and allows for adaptability in clinical practice in an evolving therapeutic landscape.

## Data Availability

All data analysed during this study are included in this published article. The data generated during the study are available in the C-BIG repository, https://www.mcgill.ca/neuro/open-science/c-big-repository.
